# Connectivity changes in two-channel prefrontal ERP associated with early cognitive decline in the elderly population: beta band responses to the auditory oddball stimuli

**DOI:** 10.3389/fnagi.2024.1456169

**Published:** 2024-10-17

**Authors:** Jang-Han Bae, Minho Choi, Jang Jae Lee, Kun Ho Lee, Jaeuk U. Kim

**Affiliations:** ^1^Digital Health Research Division, Korea Institute of Oriental Medicine, Daejeon, Republic of Korea; ^2^Aging Convergence Research Center, Korea Research Institute of Biotechnology (KRIBB), Daejeon, Republic of Korea; ^3^Asian Dementia Research Initiative, Chosun University, Gwangju, Republic of Korea; ^4^Department of Biomedical Science, Chosun University, Gwangju, Republic of Korea; ^5^Korea Brain Research Institute, Daegu, Republic of Korea; ^6^KM Convergence Science, University of Science and Technology, Daejeon, Republic of Korea

**Keywords:** mild cognitive impairment, event-related potential, two-channel prefrontal EEG, brain connectivity, synchronization, EEG beta band, auditory oddball paradigm

## Abstract

**Background:**

This study utilized recent advancements in electroencephalography (EEG) technology that enable the measurement of prefrontal event-related potentials (ERPs) to facilitate the early detection of mild cognitive impairment (MCI). We investigated two-channel prefrontal ERP signals obtained from a large cohort of elderly participants and compare among cognitively normal (CN), subjective cognitive decline (SCD), amnestic MCI (aMCI), and nonamnestic MCI (naMCI) groups.

**Methods:**

Signal processing and ERP component analyses, specifically adapted for two-channel prefrontal ERP signals evoked by the auditory oddball task, were performed on a total of 1,754 elderly participants. Connectivity analyses were conducted to assess brain synchronization, especially in the beta band involving the phase locking value (PLV) and coherence (COH). Time-frequency, time-trial, grand average, and further statistical analyses of the standard and target epochs were also conducted to explore differences among the cognition groups.

**Results:**

The MCI group’s response to target stimuli was characterized by greater response time variability (*p* < 0.001) and greater variability in the P300 latency (*p* < 0.05), leading to less consistent responses than those of the healthy control (HC) group (CN+SCD subgroups). In the connectivity analyses of PLV and COH waveforms, significant differences were observed, indicating a loss of synchronization in the beta band in response to standard stimuli in the MCI group. In addition, the absence of event-related desynchronization (ERD) indicated that information processing related to readiness and task performance in the beta band was not efficient in the MCI group. Furthermore, the observed decline in the P200 amplitude as the standard trials progressed suggests the impaired attention and inhibitory processes in the MCI group compared to the HC group. The aMCI subgroup showed high variability in COH values, while the naMCI subgroup showed impairments in their overall behavioral performance.

**Conclusion:**

These findings highlight the variability and connectivity measures can be used as markers of early cognitive decline; such measures can be assessed with simple and fast two-channel prefrontal ERP signals evoked by both standard and target stimuli. Our study provides deeper insight of cognitive impairment and the potential use of the prefrontal ERP connectivity measures to assess early cognitive decline.

## 1 Introduction

Cognitive impairment and its progression to dementia have become important public health concerns in aging populations. As life expectancy increases and the number of elderly individuals grows, the prevalence of age-related cognitive disorders has also risen (Alzheimer’s Association, 2013; Prince et al., 2015).

Mild cognitive impairment (MCI) is a transitional stage between normal cognitive decline with aging and dementia and is characterized by cognitive decline in areas such as memory, attention, language, problem-solving, and decision-making (Gauthier et al., 2006). Severe or progressive impairment can substantially impact an individual’s daily functioning and quality of life. Therefore, early detection and medical intervention are crucial for preventing cognitive decline and improving quality of life (Tisher and Salardini, 2019).

There are several subtypes of MCI, and studying the characteristics of each subtype can reveal important clinical markers regarding the risk of progression to dementia. One of the subtypes of MCI, amnestic MCI (aMCI), is characterized by substantial memory impairment, while function in the other cognitive domains is relatively preserved (Golob et al., 2007; Li et al., 2017; Yener et al., 2019). In contrast to aMCI, nonamnestic MCI (naMCI) primarily affects nonmemory cognitive functions, such as attention, executive function, and language (Corbo and Casagrande, 2022). Moreover, a growing number of individuals have reported subjective cognitive decline (SCD); despite the absence of MCI, these individuals subjectively experience memory problems (Babiloni et al., 2010). SCD is an established medical term included in the clinical practice guidelines (CPG), and SCD has been considered an intermediary stage between cognitively normal (CN) and MCI (Si et al., 2020; Lee J. S. et al., 2022). Ronnlund’s study of 2,043 individuals found that SCD was associated with a 2- to 5.7-fold increased risk of Alzheimer’s disease (AD)-related dementia, confirming a significant correlation between SCD and the onset of dementia (Ronnlund et al., 2015). The characteristics of both SCD and CN individuals, collectively referred to as healthy controls (HCs), also need to be compared with those of MCI individuals.

To assess cognitive decline and identify potential markers for early detection, neurophysiological methods utilizing bio-signals, such as electroencephalography (EEG) and event-related potentials (ERPs), have been extensively explored in MCI research (Bonanni et al., 2008; van der Flier et al., 2014; Gu and Zhang, 2017; Barry et al., 2019; Schumacher et al., 2020; Jiao et al., 2023). These techniques can directly and noninvasively measure brain activity and provide valuable insights that aid in the early detection and subtype differentiation of MCI as well as in the assessment of cognitive function during various tasks or interventions. Specifically, ERPs are electrical activities in the brain that occur in response to a specific event or stimulus. They provide a consistent and reliable pattern of neural activity evoked by auditory or visual stimuli (Gu and Zhang, 2017).

However, most previous studies have utilized multichannel EEG systems, which can be cumbersome, uncomfortable, and less practical, particularly for use in elderly individuals with cognitive decline. Older individuals generally display characteristics such as higher EEG signal variability and noise levels, as well as reduced ERP amplitudes (O’Donnell et al., 1992; O’Connell et al., 2012; K.E.S.Group, 2017), which may pose challenges for obtaining reliable measurements in real-world clinical settings.

Recent advancements in EEG technology have introduced portable and user-friendly EEG devices, such as hairband types of devices that measure activity in the prefrontal lobes using only two channels (Choi et al., 2019; Yi et al., 2019; Doan et al., 2021; Bae et al., 2022). These devices offer numerous advantages for cognitive assessment in elderly populations. Focusing on signals from a limited number of channels can achieve better control for noise and variability in the signal during measurement. Most importantly, the simplicity of this technology can enhance compliance among older populations due to convenience and quick measurement procedures.

In a study using a single channel EEG, ERPs of 15 CN subjects and 8 MCI subjects were measured with five types of auditory stimuli. This yielded ERP features in the Fpz region and enabled subsequent classification of MCI and CN subjects using random forest and support vector machine methods (Khatun et al., 2019). In another study involving 87 CN subjects and 35 individuals with dementia, a dementia prediction model was developed through a correlation analysis of two-channel prefrontal EEG and ERP biomarkers with Mini-Mental State Examination (MMSE) scores (Doan et al., 2021).

Despite the increasing availability of a few channel EEG devices, there is a paucity of research analyzing two-channel prefrontal ERP data. Moreover, prior studies of MCI using two-channel signals had several limitations.

First, there has been little discussion on how to analyze two-channel prefrontal ERP data from a signal processing perspective of elderly populations who may suffer from dementia or MCI. In a study involving 91 MCI subjects and 30 CN subjects, ERP data from the Cz and Pz regions were analyzed, revealing an increased P300 latency in MCI subjects during an auditory oddball task (Papaliagkas et al., 2008).

However, measuring data in only the left and right prefrontal lobes could result in differences in the P300 component because it is one of the most prominent ERP components in the parietal lobe. For example, it has been reported that prefrontal ERPs elicited with the oddball task had significantly smaller amplitudes of the P300 than those of the P200 and waveforms are different from those of parietal region (Winterer et al., 2003). It has also been reported that the P200 is more prominent in the frontal lobe in older individuals, while the P300 is more prominent in the parietal or occipital lobe in younger individuals (McEvoy et al., 2001).

Therefore, appropriate signal processing analyses for ERP data measured in the prefrontal lobe should be discussed. A previous study demonstrated that identifying and utilizing the optimal electrode configuration can significantly improve the accuracy of classifying MCI from CN (Lee K. et al., 2022). This implies that the results may vary based on the selected channel. However, there are limitations in applying conventional EEG analysis methods (such as independent component analysis, interpolation techniques, and rereferencing) when using two-channel device for the ease of measurement (Kiiski et al., 2012; Robbins et al., 2020). In addition, the prefrontal ERP data of elderly populations with cognitive decline often exhibit a less distinct P300 and may have different characteristics compared to typical ERP data (McEvoy et al., 2001; Winterer et al., 2003). As a result, it is necessary to utilize alternative analysis methods that comprehensively consider these issues as well as to ensure data cleaning through visual inspection with robust criteria (K.E.S.Group, 2017; Bae et al., 2022).

Second, there have been very few attempts to analyze connectivity with respect to the two-channel EEG data. Connectivity analysis of the left and right ERP signals could also provide new insights.

The phase locking value (PLV) measures the consistency of phase relationships, and coherence (COH) quantifies the degree of phase synchronization between two EEG signals at specific frequencies. These connectivity indices serve as valuable metrics for evaluating the degree of functional connectivity and synchronization of neural oscillations between different brain regions or neural populations (Burgess, 2013). The utility of the PLV and COH indices for identifying neural network and brain connectivity abnormalities associated with neurological disorders, such as dementia, AD, and epilepsy, has been discussed (Stam et al., 2003; Fonseca et al., 2011; Handayani et al., 2018).

Analysis of the PLV and COH indices offers advantages over traditional ERP component analysis in older populations. In older individuals, EEG signals often exhibit higher levels of noise and variability due to age-related changes in the brain and the aforementioned challenges in EEG measurement (O’Donnell et al., 1992; O’Connell et al., 2012). PLVs and COH values are relatively robust to noise and volume conduction, enabling researchers to extract meaningful connectivity patterns (Kaminski et al., 2016). In addition, PLVs and COH values enable frequency-specific analyses, which is crucial as age-related changes in the brain may affect neural oscillations differently across frequency bands (Vysata et al., 2012). Furthermore, while ERP component analysis focuses on the amplitude and latency of specific event-related responses, PLVs and COH values reflect functional connectivity between brain regions. These approaches offer a more comprehensive understanding of how different brain areas interact during cognitive tasks in aged population.

In such connectivity analyses, values are calculated according to different frequency bands. Among these frequency bands, the beta band is related to various cognitive functions, such as attention, memory, and information processing. The beta band is also most commonly associated with active, conscious motor behavior and sensorimotor control (Khanna and Carmena, 2017; Barone and Rossiter, 2021). Furthermore, beta rhythms are known to be involved in perceptual integration and multisensory processing (Townsend et al., 2022). While analysis of the beta range can indicate significant differences, little research has been conducted on its characteristics in two-channel prefrontal ERP data.

In a group of 20 CN subjects and 22 mild probable AD individuals, an ERP analysis of responses evoked with a visual oddball task revealed that theta waves in the F3 region in AD individuals were less phase-locked than those in CN individuals (Yener et al., 2007). In another ERP study involving 21 CNs and 22 MCI patients, a larger delta response was observed in CN subjects during the auditory oddball task (Kurt et al., 2014).

Third, a detailed analysis of each ERP evoked by standard and target stimuli is currently lacking. Responses to auditory stimuli can significantly differ depending on whether the stimuli are classified as standard (frequent or expected events) or target (rare or unexpected events) (Barry et al., 2000). The physiological and neurological explanations for these responses are grounded in our brain’s inherent bias for novelty and its capacity for predictive coding (Winkler and Czigler, 2012). Responses to standard stimuli can reflect habituation, sensory processing, and baseline neural activity, while responses to target stimuli can be used to investigate neural mechanisms related to attentional processes and the cognitive evaluation of novel or infrequent events (Skosnik et al., 2007). While ERP difference waves are useful for isolating target-specific neural activity, analyzing the ERPs evoked by standard and target stimuli separately can provide richer and more valuable insights into the underlying neural processes. Analyses should incorporate diverse perspectives, such as time-frequency analysis and time-trial analysis, as well as ERP component and connectivity analysis.

Fourth, most previous studies have conducted research in a laboratory environment and included small number of participants, which makes it challenging to consider clinical applications of the findings (Yener et al., 2007; Papaliagkas et al., 2008; Papadaniil et al., 2016; Khatun et al., 2019). Since it is difficult to replicate the laboratory environment in real-world contexts (such as clinical practice), analyzing ERP responses in actual clinical studies, which have a larger and more diverse population, is a more reliable way to determine their potential clinical relevance and usefulness. Additionally, previous studies have only compared MCI and CN groups without including more detailed comparisons of data from aMCI, naMCI, and SCD subgroups.

To address the four limitations outlined in the preceding paragraphs, the present study aimed to investigate and compare two-channel prefrontal ERP signals obtained from numerous elderly participants in a clinical study, subdivided into the CN, SCD, aMCI, and naMCI subgroups. In particular, we focused on analyzing the response characteristics evoked by standard and target stimuli in the beta band, which has been implicated in various cognitive processes and connectivity within the brain.

## 2 Materials and methods

### 2.1 Participants and experimental design

**FIGURE 1:**
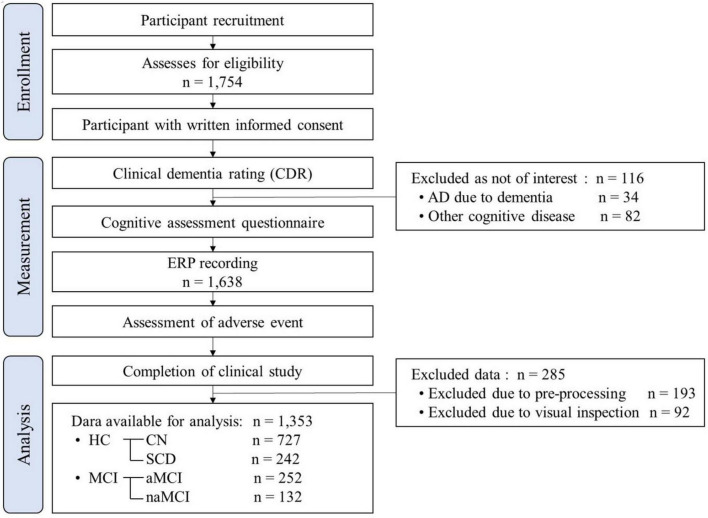
Experimental workflow in accordance with the Strengthening the Reporting of Observational Studies in Epidemiology (STROBE) Statement. HC, healthy control; CN, cognitively normal; SCD, subjective cognitive declines; MCI, mild cognitive impairment; aMCI, amnestic mild cognitive impairment; naMCI, non-amnestic mild cognitive impairment.

The participants aged 54 to 90 years were recruited from the Gwangju Alzheimer’s and Related Dementia (GARD) cohort in South Korea from 2019 to 2022. The experimental protocol was approved by the Ethics Committee of the Institutional Review Board of Chonnam National University Hospital, South Korea, and the approval number was CNUH-2019-279. This trial was conducted in accordance with the principles of the Declaration of Helsinki. Participants were recruited through phone calls, brochures, flyers, and poster advertisements and were asked to sign informed consent forms after receiving a full explanation of the study. Experimental workflow in accordance with the Strengthening the Reporting of Observational Studies in Epidemiology (STROBE) Statement is shown in Figure 1.

This study analyzed ERP data from 1,754 participants, all of whom underwent a clinical interview and were assigned a Clinical Dementia Rating (CDR) score. In addition, all individuals underwent cognitive function evaluations using several cognition test tools (Park et al., 2019): the Korean version of the MMSE (K-MMSE), which is used to measure global cognitive decline and has simple 30 questions; the Seoul Neuropsychological Screening Battery II (SNSB II), which encompasses five cognitive domains, including attention, language, memory, visuospatial abilities, and frontal/executive function; the Korean Dementia Screening Questionnaire-Cognition (KDSQ-C), which is known for its sensitivity in detecting early-stage dementia; the Korean version of the Geriatric Depression Scale (K-GDS), which is to evaluate geriatric depression; and the Korean version of the Instrumental Activities of Daily Living scale (K-IADL), which is used to assess participants’ daily functioning.

All participants were divided into 2 main groups: the HC and MCI groups. These groups were further divided into 4 subgroups: CN and SCD subgroups (within the HC group) as well as the aMCI and naMCI subgroups (within the MCI group). If the CDR score was 0, the participant was placed into the HC group and then undergoes a clinical evaluation by a physician. If the primary symptom reported is subjective memory complaints, the physician diagnosed participants with SCD based on the existing CPG and a comprehensive evaluation of various clinical factors (Si et al., 2020; Lee J. S. et al., 2022). Meta-analysis indicates that the CDR is an effective tool for staging cognitive decline, with cutoff points of 0.5 and ≥ 1.0 used to screen for MCI and dementia, respectively (Huang et al., 2021). Therefore, a CDR score of 0.5 was considered to reflect MCI, except for participants who met the following criteria: (1) two or more white matter hyperintensities (WMHs) on magnetic resonance (MR) images, (2) a GDS score of 17 or more, (3) cerebral diseases such as stroke or cerebral hemorrhage, and (4) internal medical conditions that may affect cognitive function. In addition, participants were placed in the aMCI subgroup if their z scores in the memory domain of the SNSB II were below −1.5 (Park et al., 2019). On the other hand, participants were placed in the naMCI subgroup if they had z scores of −1.5 or less in at least one of the other four domains. Otherwise, participants with CDR scores greater than 0.5 were classified having dementia.

### 2.2 ERP recording using a portable device

ERP signals were measured using a portable device, NeuroNicle FX2, which was manufactured by LAXTHA in Daejeon, Republic of Korea. The device has been widely used in many hospitals, public institutions and centers for dementia, and its reliability and that of the calculated EEG variables have been confirmed in previous studies (Choi et al., 2019). In addition, the device is also optimized for simple and easy measurement. For ERP recording, two noninvasive monopolar scalp electrodes were used to measure the EEG signals of the prefrontal regions, specifically Fp1 and Fp2, according to the international 10/20 electrode system. The reference electrode was placed on the right earlobe. To ensure signal quality, digital infinite impulse response Butterworth filters were applied to the device. These included a 2nd-order band-stop filter with a range of 55 Hz to 65 Hz to eliminate power line noise, a 1st-order high-pass filter with a cutoff frequency of 2.6 Hz to remove low-frequency drift, and an 8th order low-pass filter with a cutoff frequency of 43 Hz to attenuate high-frequency noise (Widmann et al., 2015). The input range was ± 393 μV with 0.6 μV or less input noise, and all contact impedances were kept below 10 kΩ. The ERP signals were acquired at a sampling rate of 250 Hz with 15-bit resolution. Our research team previously analyzed the reliability of EEG signals at the Fp1 and Fp2 regions using the same EEG equipment employed in the present study (Choi et al., 2019).

During recording, participants sat in an upright seated position with their eyes closed for a duration of 5 min and 21 s. To minimize artifacts caused by blinking, muscle movement, and external noise, trained clinical research nurses closely monitored the participants and the ERP signals in a quiet room with regular illumination. If any events occurred, such as participant movement, dozing off, or external noise, the nurses notified the participant, and the corresponding portion of the ERP signal was excluded from the analysis. Our data are available upon request to facilitate replication of our findings.

### 2.3 Task and stimuli

In this study, we employed an active auditory oddball task to elicit selective-attention ERPs. Prior to the experiment, all participants underwent hearing ability tests. The oddball task consisted of 256 monotonic standard auditory stimuli at 750 Hz and 64 rare randomly distributed target auditory stimuli at 2,000 Hz. The ratio of the two stimulus types was set at 4:1, and their intensity was maintained at 80 dB. Each stimulus had a duration of 50 ms, and the interstimulus interval was set at 1,000 ms. Participants were instructed to press a designated response key upon recognizing the target stimuli. The time indices corresponding to the occurrence of the standard or target stimuli, as well as the time indices of participants’ responses, were recorded separately for subsequent calculation of behavioral measures.

In this study, we calculated the number of correct responses and the error rate from both data recorded in standard trials (standard epochs) and data recorded in target trials (target epochs). Additionally, the average response time and its standard deviation (SD) were computed for correction behavioral responses for target stimuli (see Supplementary Table 1 for more details).

### 2.4 Pre-processing of two-channel ERP signals from the prefrontal lobe

The prefrontal two-channel ERP data obtained from elderly individuals not only are of poor quality but also consist of waveforms with small amplitudes. As a result, applying conventional EEG analysis approaches is difficult. Methods such as principal component analysis or independent component analysis, commonly used for EEG noise removal, cannot be directly applied. Interpolation techniques or rereferencing based on common averaging references are also not viable options (Kiiski et al., 2012; Robbins et al., 2020). Furthermore, the P300 component, which is typically prominent in the parietal lobe, may exhibit different characteristics in the prefrontal lobe.

To address these issues, we developed a preprocessing and ERP component extraction method suitable for use with two-channel ERP data obtained from the prefrontal lobe of elderly individuals. This method builds upon our previous work (Bae et al., 2022). The developed signal preprocessing methods included filtering, epoching, baseline correction, calculation of response error rate, artifact rejection, random selection, and averaging. A 0.1∼30 Hz finite impulse response bandpass filter was applied to reduce noise components in the high frequency and low frequency band. All recorded ERP signals underwent visual inspection, and data contaminated by eye or muscle noise, as well as unexpected external signals, were excluded from the analysis. More detailed information on each pre-processing method can be found in the Supplementary material. A total of 193 participants were automatically excluded in data pre-processing steps, and data from an additional 92 participants were excluded based on visual inspection criteria.

For the pre-processing of ERP signals and ERP components as well as connectivity, time-frequency (TF), time-trial (TT), grand average, and further statistical analyses, an ERP analysis program was developed in MATLAB R2023a based on EEGLAB functions.

### 2.5 ERP component analyses

The proposed method for ERP component extraction begins with optimizing the search range or time window to identify the ERP components. Given that in the prefrontal regions, the P200 component may be more prominent than the P300 component and that the waveform characteristics in this region may differ from the typical ERP waveforms frequently observed in the parietal lobe, it was necessary to define appropriate time windows for component analysis.

Previous reports of smaller ERP amplitudes and slower latencies in older adults were considered during a visual inspection of the ERP signals recorded in this study; we observed that the P200 component often lasted up to 300 ms, while the P300 component rarely appeared before 300 ms. Given these observations, we set the time windows as follows: 60∼200 ms for the N100, 180∼300 ms for the P200, and 300∼600 ms for the P300. To ensure the reliability of the identified peaks, a minimum amplitude threshold of 2 μV was set. Peaks smaller than this threshold or those detected at the boundaries of the time window were not considered ERP peaks. Notably, even if the P300 was not clearly visible, the analysis continued if the N100 or P200 components were present in the target ERP data.

In this study, ERP waveforms were generated by averaging across all epochs for each dataset. The latencies and amplitudes of each of the N100, P200, and P300 components were then calculated. Additionally, the mean amplitude of the P300 component, as well as the SDs of the P300 latencies and amplitudes across trials, were computed (see Supplementary Table 1 for more details). All ERP waveforms and components were obtained separately for both the standard and target epochs.

### 2.6 ERP connectivity analyses

The PLV is a widely used index to measure the strength of phase synchronization in neural activity, and it has the advantage of being robust to signal noise and volume conduction. ERPs are transient EEG signals observed in response to specific stimuli and are thus susceptible to noise and variability. The PLV enables a direct examination of ongoing phase dynamics and synchronization, offering a complementary perspective to ERP component-based analyses.

To calculate the PLV, a finite-impulse response bandpass filter was first applied to the ERP signals in the frequency domain of interest, followed by the application of the Hilbert transform to independently extract the phase component from each channel and calculate the phase difference between them. Finally, the PLV between two signals was defined as the average value, as shown in Equation 1.


(1) $$P\,L\,V\,\left(t\right)=\frac{1}{N}\,\left|\sum_{n=1}^{N}e\,x\,p\,\left(j\,\theta \,\left(t,n\right)\right)\right|$$

where θ is the phase difference at a specific time t of the nth epoch, and N is the total number of epochs. The PLV reflects the degree of phase synchronization on a scale of 0 to 1, where 0 represents no synchronization and 1 represents perfect synchronization.

In this study, PLV waveforms over time were generated for both standard and target epochs in the 4∼8 Hz for theta, 8∼13 Hz for alpha, and 13∼30 Hz for beta frequency bands. In addition, the maximum value of the PLV and the corresponding time at which it occurred and the mean PLVs within the P200, P300 and positive time windows were calculated for each of the three frequency bands (see Supplementary Table 1 for more details).

The COH is an index that reflects the consistency or variability of the phase angles between two time series, taking different frequencies into account (Thatcher et al., 2005). In this study, COH analysis focused on two channels in the prefrontal lobe; thus, the potential impact of electrode distance on COH values was not considered (Fonseca et al., 2011). The COH is defined as the square cross-spectrum of the signals from two channels divided by the product of the power spectral densities (PSDs) of each signal, as shown in Equation 2.


(2) $$C_{x\,y}\,\left(f\right)=\frac{{\left|W_{x\,y}\right|}^{2}\,\left(f\right)}{W_{x\,x}\,{\left(f\right)}^{*}\,W_{y\,y}\,\left(f\right)}$$

where W_*xy*_ is the cross-spectral density of the two signals, W_*xx*_ is the PSD of x, W_*yy*_ is the PSD of y, and f is the frequency, and C_*xy*_ (f) is the COH between x and y channels at frequency f. A value of 0 means there is no linear dependence between x and y at frequency f.

In this study, the COH waveforms over frequency for both standard and target epochs in the theta, alpha, and beta frequency bands were generated. In addition, the maximum value of the COH and the corresponding frequency at which it occurred and the means and SDs of the COH values were calculated for each of the three frequency bands. Furthermore, the mean and SD of COH values across all frequency bands were calculated by Welch’s averaged periodogram method, and this calculation was performed using the open source software toolbox HERMES for MATLAB (see Supplementary Table 1 for more details).

### 2.7 Time-frequency and time-trial analyses

For the TF analysis of the ERP signal, event-related spectral perturbation (ERSP) and inter-trial coherence (ITC) were calculated based on the fast Fourier transform with a window size of 64 using Hanning tapers.

ERSP was calculated by comparing brainwave activity across frequency bands during two specific periods. The first period was the baseline subwindow from 200 ms before the onset of the stimulus to the moment the stimulus was presented (−200 to 0 ms relative to stimulus onset), and the second period was the period after stimulus onset ( > 0 ms relative to stimulus onset). ERSP analysis provides information about the frequency power present at specific time points. If ERSP is increased compared to that in the baseline period, it is classified as event-related synchronization (ERS), and if ERSP is decreased, it is classified as event-related desynchronization (ERD).

ITC was calculated by averaging the phase information in the frequency domain across multiple trials. It provides a measure of the degree of periodicity or correlation at a specific frequency and time interval (Basar et al., 2015). ITC provides a direct indication of the degree of phase synchronization, with a value of 1 indicating perfect synchronization between the signals. In this study, TF plots of ERSP and ITC were generated as color maps for both the standard and target epochs. Furthermore, permutation analysis of the baseline subwindow was performed to identify areas exhibiting significant differences.

Additionally, the ERP data were visualized from a temporal perspective to identify the brainwave activity associated with an event in the TT analysis of ERP signals. TT analysis provides insights into temporal changes, patterns, and significant fluctuations associated with the event, providing a comprehensive understanding of the dynamic nature of brain responses. In this study, TT plots of the ERP values were generated as color maps for both the standard and target epochs.

### 2.8 Grand average analyses

Grand average analyses enable visual identification of macro changes in each group with greater confidence and detail. First, each ERP waveform, obtained through the proposed signal processing method, was averaged separately for the 2 main groups and 4 subgroups to generate the grand average ERP waveforms. Next, grand average PLV waveforms were generated over time for each of the 4 subgroups in the theta, alpha, and beta bands. Subsequent analyses examined significant differences between the 2 main groups based on the comparison of averages across 100 ms intervals (0∼700 ms). Additionally, to gain a macroscopic perspective considering all values from the grand average waveform shape, we examined all time points where significant differences between the 2 main groups. This detailed analysis allowed a more comprehensive examination of the temporal changes over the specified period.

In a similar manner, grand average COH waveforms over frequency were generated for each of the 4 subgroups. Subsequent analyses examined significant differences between the 2 main groups based on the comparison of averages across seven frequency ranges (4∼8 Hz for theta, 8∼10 Hz for low alpha, 10∼13 Hz for high alpha, 13∼16 Hz for low beta, 16∼20 Hz for middle beta, 20∼23 Hz for high beta and 23∼30 Hz for very high beta band). Additionally, all frequency ranges were analyzed to identify significant differences between the 2 main groups to comprehensively examine the frequency-specific differences between the groups. In addition, grand average TF plots of ERSP and ITC, as well as grand average TT plots, were also generated across the 4 subgroups and 2 main groups by averaging the values from the two channels. For the color maps representing the grand averages of ERSP and ITC values, significant values with a permutation statistics p value below 0.01 are indicated in colors, while nonsignificant values are plotted in green. For the grand average plots of TT, sorted epochs with a 10-epoch moving window were applied for smoothing.

### 2.9 Statistical analyses

The demographic and neuropsychological measures for the 4 subgroups are summarized using means and SDs. The analyzed variables, including behavioral measures, ERP components, and connectivity, are summarized in Supplementary Table 1.

To compare the mean differences of these variables between the HC and MCI groups, independent-sample t tests were conducted after checking the normality of the data with the Shapiro-Wilk test and equal variance test. In cases where Levene’s test for equal variance was violated, Welch’s t tests were employed. In addition, ANCOVAs were conducted to analyze the between-group differences while controlling for potential effects of covariates, including age, sex, and education level.

Furthermore, the mean differences among the 4 subgroups were analyzed using ANCOVA with the same three covariates mentioned above, employing a parallel lines model. Post hoc tests were conducted using Bonferroni’s method to further examine specific group differences. In cases where variables exhibited abnormal distribution, a logarithmic transformation was applied. The threshold of significance for all statistical tests was set to α = 0.05.

## 3 Results

### 3.1 Demographic and neuropsychological characteristics

Table 1 shows the demographic and neuropsychological characteristics, including scores on the five cognitive domains of the SNSB II, the K-MMSE, the K-GDS, the KDSQ-C and the K-IADL, of the 2 main groups and 4 subgroups in terms of the means and SDs. There were 1,353 total participants (CN = 727, SCD = 242, aMCI = 252, and naMCI = 132). The HC group had 969 participants (523 males and 446 females), and their mean age was 72.07 years; the MCI group had 384 participants (232 males and 152 females), and their mean age was 73.86 years (*p* < 0.01). The MCI group exhibited lower SNSB II scores in the five cognitive domains and lower K-MMSE scores than the HC group (*p* < 0.01). The KDSQ-C, K-GDS and K-IADL scores showed no statistically significant differences between groups.

**TABLE 1:** Demographic and neuropsychological characteristics of the 2 main groups and 4 subgroups.

	HC (*n* = 969)	MCI (*n* = 384)	*p*-value	CN (*n* = 727)	SCD (*n* = 242)	aMCI (*n* = 252)	naMCI (*n* = 132)
Age (years)	72.07 ± 6.21	73.86 ± 6.66	*p* < 0.001	72.32 ± 6.19	71.34 ± 6.21	74.26 ± 6.79	73.10 ± 6.36
Sex (male, %)	523, 53.97	232, 60.42	0.034 (χ^2^ = 4.630)	415, 57.08	108, 44.63	163, 64.68	69, 52.27
Education level (years)	11.87 ± 4.57	12.36 ± 4.70	0.077	11.80 ± 4.60	12.11 ± 4.51	12.20 ± 4.72	12.68 ± 4.68
Attention score	9.71 ± 2.13	8.57 ± 2.02	*p* < 0.001	9.67 ± 2.10	9.80 ± 2.20	8.66 ± 1.92	8.41 ± 2.20
Language score	0.20 ± 0.29	−0.09 ± 0.45	*p* < 0.001	0.19 ± 0.30	0.21 ± 0.26	−0.08 ± 0.45	−0.11 ± 0.44
Visuospatial score	0.51 ± 0.43	0.16 ± 0.74	*p* < 0.001	0.49 ± 0.45	0.57 ± 0.32	0.15 ± 0.77	0.18 ± 0.66
Memory score	0.27 ± 0.57	−0.49 ± 0.62	*p* < 0.001	0.26 ± 0.56	0.29 ± 0.59	−0.74 ± 0.54	0.01 ± 0.47
Frontal/executive score	0.25 ± 0.56	−0.22 ± 0.66	*p* < 0.001	0.24 ± 0.56	0.27 ± 0.57	−0.23 ± 0.67	−0.18 ± 0.64
K-MMSE score	27.78 ± 1.86	26.42 ± 2.72	*p* < 0.001	27.78 ± 1.84	27.76 ± 1.92	26.23 ± 2.74	26.78 ± 2.65
KDSQ-C score	3.24 ± 2.49	3.28 ± 2.79	0.797	3.10 ± 2.40	3.66 ± 2.72	3.37 ± 2.81	3.11 ± 2.75
K-GDS score	8.17 ± 6.72	8.68 ± 6.71	0.208	7.65 ± 6.45	9.76 ± 7.26	8.92 ± 6.82	8.23 ± 6.52
K-IADL score	0.05 ± 0.27	0.06 ± 0.29	0.736	0.06 ± 0.28	0.05 ± 0.25	0.08 ± 0.35	0.02 ± 0.10

The scores for attention, language, visuospatial, memory and frontal/executive functions were derived from the Seoul Neuropsychological Screening Battery II (SNSB II). K-MMSE, Korean version of the Mini-Mental State Examination; KDSQ-C, Korean Dementia Screening Questionnaire-Cognition; K-GDS, Korean version of the Geriatric Depression Scale; and K-IADL, Korean version of the Instrumental Activities of Daily Living scale.

### 3.2 Comparison of the mean differences

Table 2 summarizes the mean differences in the analyzed variables, including behavioral measures, ERP components and connectivity, between the HC and MCI groups for each standard and target epoch.

**TABLE 2:** Mean differences in the analyzed variables between the HC and MCI groups for each standard and target epoch.

Variable	Standard				Target			
	**HC**	**MCI**	***p*-value^a^**	***p*-value^b^**	**HC**	**MCI**	***p*-value^a^**	***p*-value^b^**
Cor_no	254.13 ± 2.78	253.47 ± 3.70	0.002**	0.002**	62.54 ± 2.70	62.09 ± 2.98	0.011*	0.016*
Err_rate	0.73 ± 1.09	0.99 ± 1.45	0.002**	0.002**	2.29 ± 4.22	2.98 ± 4.66	0.011*	0.012*
RT_mean	–	–	–	–	343.60 ± 63.91	344.55 ± 65.49	0.807	0.542
RT_std	–	–	–	–	78.08 ± 19.36	82.26 ± 19.99	0.001**	0.001**
N100_lat	147.19 ± 16.48	147.14 ± 14.43	0.959	0.864	144.05 ± 23.46	143.96 ± 22.86	0.956	0.943
N100_amp	±3.57 ± 1.32	±3.60 ± 1.31	0.745	0.752	−4.05 ± 1.73	−4.10 ± 1.80	0.641	0.422
P200_lat	244.60 ± 32.45	244.72 ± 34.75	0.965	0.744	251.16 ± 25.92	252.61 ± 22.52	0.343	0.432
P200_amp	3.29 ± 1.69	3.25 ± 1.34	0.670	0.502	6.09 ± 3.77	6.00 ± 3.53	0.702	0.827
P300_lat	435.09 ± 92.48	438.25 ± 92.57	0.603	0.315	397.28 ± 75.24	404.69 ± 76.82	0.127	0.194
P300_lat_std	79.04 ± 16.62	80.83 ± 18.00	0.110	0.104	52.13 ± 25.00	55.45 ± 26.89	0.043*	0.036*
P300_amp	3.21 ± 3.15	3.28 ± 3.18	0.733	0.924	7.64 ± 7.21	7.89 ± 6.72	0.580	0.640
P300_amp_std	2.71 ± 1.45	2.81 ± 1.50	0.310	0.291	2.95 ± 2.02	2.99 ± 2.01	0.732	0.616
P300_amp_mean	0.49 ± 1.80	0.61 ± 1.94	0.281	0.736	1.25 ± 4.01	1.52 ± 3.52	0.232	0.348
PLV_max_B	0.73 ± 0.15	0.71 ± 0.15	0.054	0.053	0.80 ± 0.13	0.78 ± 0.13	0.078	0.060
PLV_time_B	310.82 ± 202.99	308.25 ± 203.94	0.834	0.747	299.30 ± 188.46	315.25 ± 189.60	0.161	0.192
PLV_P200_B	0.65 ± 0.16	0.63 ± 0.17	0.057	0.056	0.66 ± 0.16	0.64 ± 0.17	0.069	0.058
PLV_P300_B	0.64 ± 0.16	0.62 ± 0.17	0.058	0.053	0.64 ± 0.16	0.63 ± 0.17	0.173	0.146
PLV_P_B	0.64 ± 0.16	0.62 ± 0.17	0.051	0.049*	0.65 ± 0.16	0.63 ± 0.17	0.101	0.092
COH_max_B	0.71 ± 0.17	0.69 ± 0.17	0.179	0.180	0.74 ± 0.16	0.72 ± 0.16	0.143	0.112
COH_freq_B	16.30 ± 4.35	16.43 ± 4.70	0.627	0.739	16.93 ± 4.70	16.88 ± 4.57	0.869	0.833
COH_mean_B	0.55 ± 0.21	0.53 ± 0.21	0.099	0.077	0.56 ± 0.21	0.54 ± 0.21	0.216	0.150
COH_std_B	9.19 ± 5.82	9.48 ± 5.80	0.406	0.268	10.18 ± 5.62	10.16 ± 5.36	0.964	0.774
COH_mean	0.63 ± 0.17	0.61 ± 0.17	0.169	0.157	0.64 ± 0.17	0.63 ± 0.17	0.212	0.170
COH_std	8.78 ± 4.03	9.45 ± 4.22	0.007**	0.006**	9.88 ± 4.15	10.42 ± 4.29	0.032*	0.023*

^a^Obtained from an independent-sample *t*-test.

^b^Obtained from ANCOVA that included age, sex and education level as covariates. RT_mean and RT_std were computed in only the target epoch. The connectivity variables in this table show only values calculated from the beta band.

**P < 0.01;

*P < 0.05.

The results of independent-sample t tests showed that Cor_no, Err_rate and COH_std (*p* < 0.01) in the standard epoch and Cor_no, Err_rate, P300_lat_std, COH_std (p < 0.05) and RT_std (*p* < 0.01) in the target epoch significantly differed between the groups. The ANCOVA results showed that all the variables that significantly differed in independent-sample t tests also showed significant differences in the ANCOVAs. In addition, a significant difference was found in PLV_P_B from the beta band in the standard epoch. Neither standard nor target epochs showed significant differences in the connectivity variables calculated in the alpha and theta bands.

**FIGURE 2:**
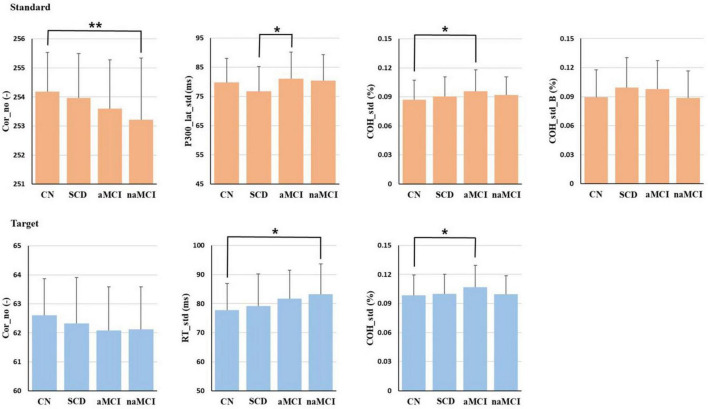
The ANCOVA results of the 4 subgroups. Only the variables that were analyzed and showed significant differences are displayed. Age, sex, and education level were used as covariates, and the Bonferroni method was employed for post-hoc analysis. *p < 0.05, **p < 0.01.

The results of ANCOVA including only variables with significant differences using three covariates for comparing mean differences among the 4 subgroups are presented in Figure 2. In the standard epoch, Cor_no showed significant differences (*p* < 0.01) among the 4 subgroups, with the order being CN > SCD > aMCI > naMCI. Additionally, COH_std showed significant differences (*p* < 0.05) among the 4 subgroups, with the order being aMCI > naMCI > SCD > CN. Furthermore, P300_lat_std and COH_std_B also demonstrated significant differences (*p* < 0.05) among the 4 subgroups. According to the results of Bonferroni’s post hoc test, Cor_no was significantly higher in the CN subgroup than in the naMCI subgroup (*p* < 0.01), P300_lat_std was significantly higher in the aMCI subgroup than in the SCD subgroup (*p* < 0.05), and COH_std was significantly lower in the CN subgroup than in the aMCI subgroup (*p* < 0.05).

In the target epoch, Cor_no showed significant differences (*p* < 0.05) among the 4 subgroups, and Cor_no for SCD was smaller than that for CN but larger than that for the MCI group. In addition, RT_std revealed significant differences (*p* < 0.01) among the 4 subgroups, with CN exhibiting the smallest variability. SCD showed greater variability than CN but less than the MCI group. Furthermore, COH_std showed significant differences (*p* < 0.05) among the 4 subgroups, with the order being aMCI > naMCI > SCD > CN. According to the results of Bonferroni’s post hoc test, RT_std was significantly lower in the CN subgroup than in the naMCI subgroup (*p* < 0.01), and COH_std was significantly lower in the CN subgroup than in the aMCI subgroup (*p* < 0.05).

### 3.3 Grand average ERP waveforms

**FIGURE 3:**
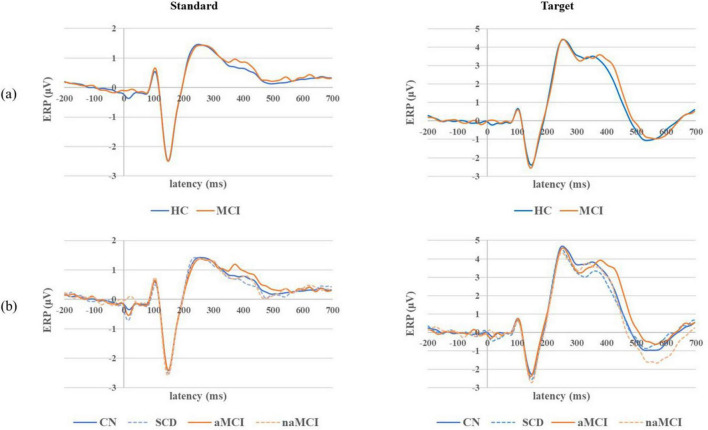
Grand average ERP waveforms for both standard and target epochs in the **(a)** 2 main groups and **(b)** 4 subgroups.

Grand average ERP waveforms for both standard and target stimuli in the 2 main groups and 4 subgroups are shown in Figure 3. In response to standard stimuli, the N100 component was the most dominant, and the P200 component was also noticeable, but the P300 component was rarely seen. In response to target stimuli, the N100, P200 and P300 components were clearly noticeable; however, in contrast to the results of the individual ERP component analyses, the P200 amplitude was slightly larger than the P300 amplitude. In addition, the P200 component was similar between the 2 main groups, but the P300 latency was slightly longer in the MCI group than in the HC group. In terms of the responses of the 4 subgroups, the P300 latency tended to be longer in the aMCI subgroup than in the other three groups.

### 3.4 Grand average waveforms for connectivity analyses

**FIGURE 4:**
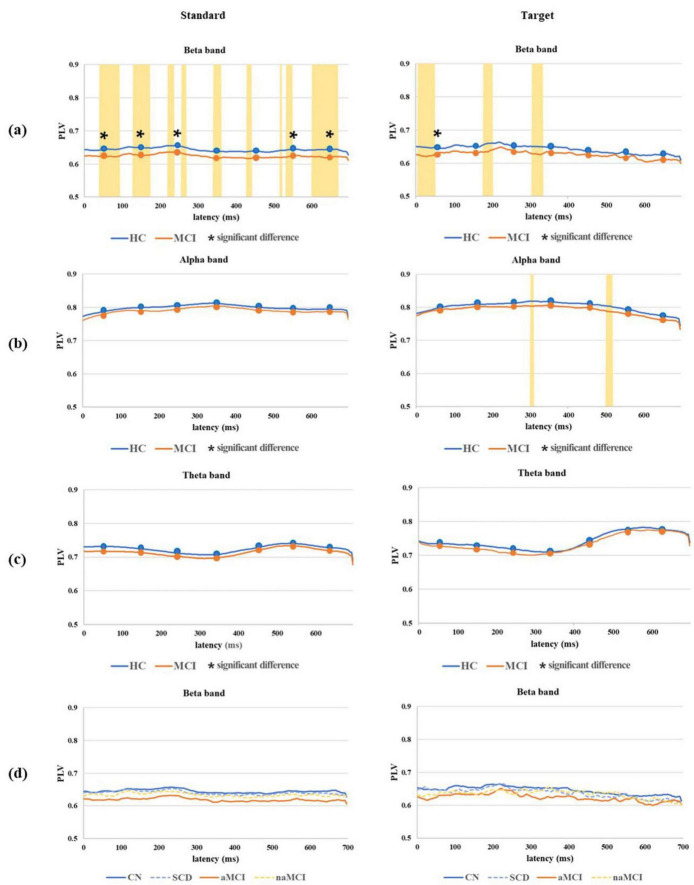
Grand average PLV waveforms for both standard and target epochs in the **(a)** beta band, **(b)** alpha band, and **(c)** theta band with significant difference time intervals (marked with an asterisk) based on the comparison of averages across seven 100 ms intervals between the 2 main groups and in the **(d)** beta band among the 4 subgroups. All time points on the grand average PLV waveform, where significant differences between the 2 main groups were observed, are marked by the yellow area. *P < 0.05.

Grand average PLV waveforms for both the standard and target epochs in the beta, alpha and theta bands with significant difference time intervals between the 2 main groups and among the 4 subgroups are shown in Figure 4. For the standard PLV waveforms in the beta band, significant differences based on the comparison of averages between the HC and MCI groups were observed (0∼300 ms and 500∼700 ms). Furthermore, significant differences from the analysis across all time points were evenly distributed throughout the entire time range. In the analysis of the 4 subgroups, PLVs were highest throughout the entire time range in the CN subgroup, followed by SCD, naMCI, and aMCI. On the other hand, PLV waveforms in the theta and alpha bands were not significantly different between the HC and MCI groups. For the target PLV in the beta band, significant differences were observed between the HC and MCI groups based on the comparison of averages within the 0∼100 ms interval. However, no significant difference was found in the alpha and theta bands. In the 4 subgroups, both standard and target PLVs were the highest in the CN subgroup and the lowest in the aMCI subgroup.

**FIGURE 5:**
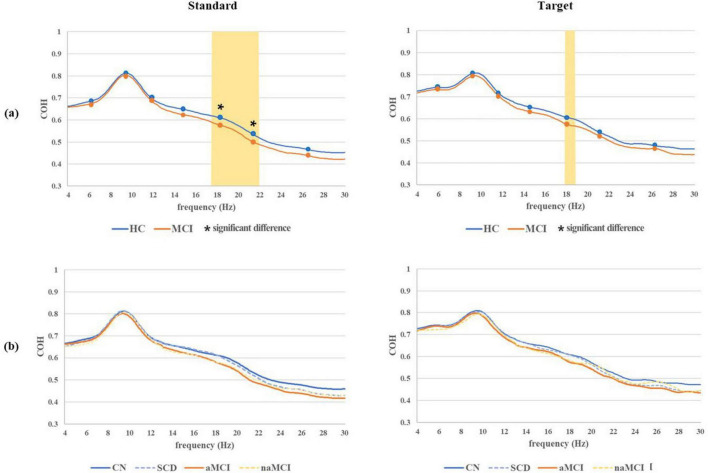
Grand average COH waveforms for both standard and target epochs with significant difference frequency ranges (marked with an asterisk) based on the comparison of averages across seven frequency ranges between the **(a)** 2 main groups and **(b)** 4 subgroups. All frequency ranges on the grand average COH waveform, where significant differences between the 2 main groups were observed, are marked by the yellow area. *P < 0.05.

Grand average COH waveforms for both the standard and target with significant difference frequency ranges in the 2 main groups and 4 subgroups are shown in Figure 5. In terms of the standard COH waveforms, the MCI group had lower values than the HC group overall, and significant differences in COH waveforms were found in the region containing the middle and high beta bands. In the analysis of the 4 subgroups, COH values were highest in the CN subgroup, followed by SCD, naMCI, and aMCI. However, the theta and alpha bands showed no significant group differences. In terms of the target COH waveforms, the MCI group also had lower values than the HC group overall, but no significant difference based on the comparison of averages between the HC and MCI groups was found. In the 4 subgroups, both standard and target COH values were the highest in the CN subgroup and the lowest in the aMCI subgroup.

### 3.5 Grand average for TF and TT analyses

**FIGURE 6:**
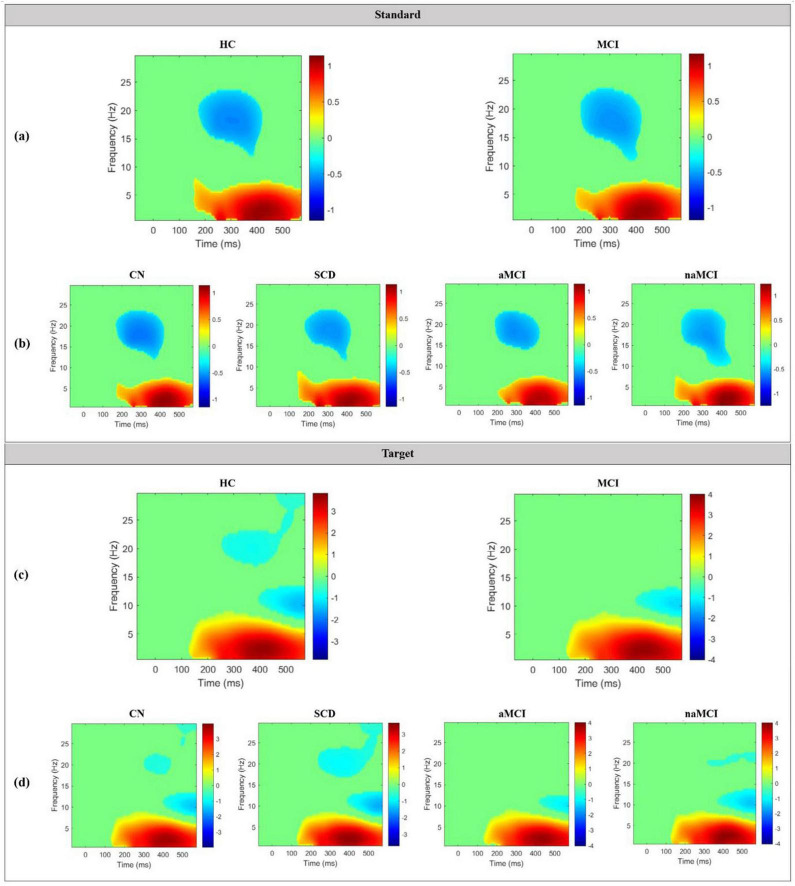
Grand average TF plots of ERSP for standard epochs in the **(a)** 2 main groups and **(b)** 4 subgroups, and for target epochs in the **(c)** 2 main groups and **(d)** 4 subgroups. Significant values compared to the baseline window are indicated in colors, while non-significant values are plotted in green.

Grand average TF plots of ERSP for both standard and target epochs in the 2 main groups and the 4 subgroups are shown in Figure 6. In the standard TF plots of ERSP in the 2 main groups, ERS occurred in the delta and theta bands from approximately 170 to 550 ms, and ERD occurred in the beta bands from approximately 200 to 400 ms. Among the 4 subgroups, only the aMCI subgroup showed a slightly different trend than the other groups, with no ERD in the approximately 10∼14 Hz range between approximately 320 and 400 ms. In the target TF plots of ERSP, ERS occurred in the delta and theta bands at approximately 120–550 ms in both the HC and MCI groups. However, ERD was mainly seen in the middle and high beta band between approximately 300 and 500 ms in the HC group but not in the MCI group. Notably, this trend was more pronounced in the aMCI subgroup than in the naMCI subgroup.

**FIGURE 7:**
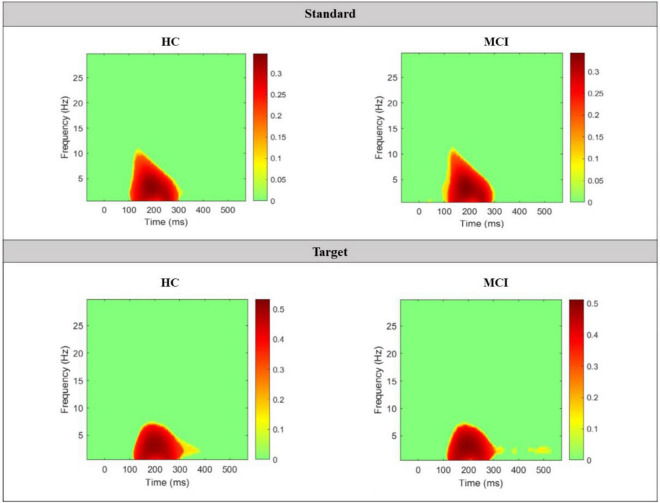
Grand average TF plots of ITC for both standard and target epochs in the 2 main groups. Significant values compared to the baseline window are indicated in colors, while non-significant values are plotted in green.

Grand average TF plots of ITC for both standard and target epochs in the 2 main groups are shown in Figure 7. In terms of the standard TF plots of ITC, both the HC and MCI groups had similar significant differences in the delta and theta bands between approximately 100 and 300 ms, with an ITC value of more than 0.3, and in the alpha bands at approximately 150 ms. Similarly, in the target TF plots of ITC, significant differences were found in the delta and theta bands between 100 and 300 ms with an ITC value of approximately 0.5.

**FIGURE 8:**
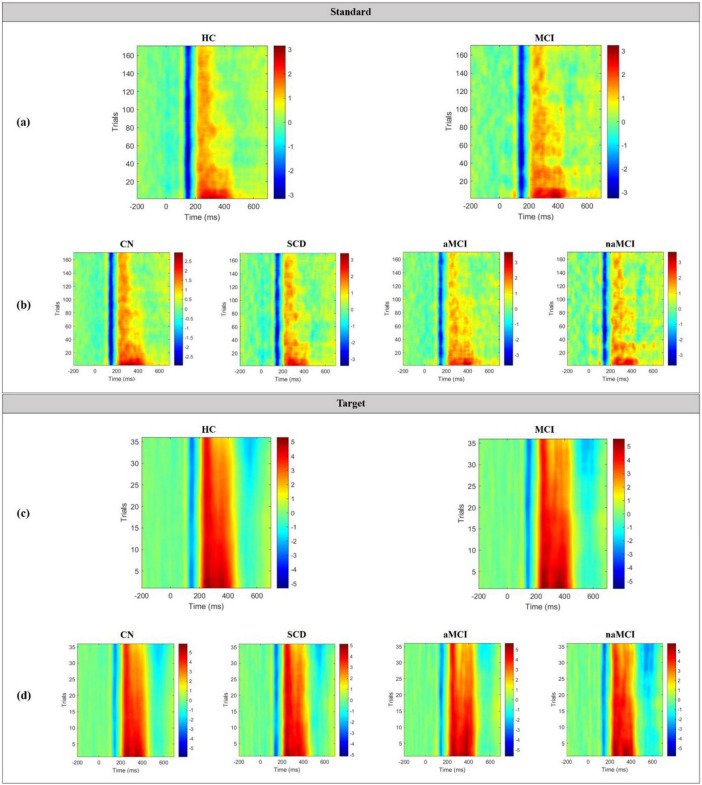
Grand average TT plots for standard epochs in the **(a)** 2 main groups and **(b)** 4 subgroups, and for target epochs in the **(c)** 2 main groups and **(d)** 4 subgroups.

Grand average TT plots for both standard and target epochs in the 2 main groups and 4 subgroups are shown in Figure 8. In the standard TT plots of both groups, the N100 component occurred at approximately 150 ms with a maximum amplitude of approximately −3 μV, and the P200 component occurred at approximately 200 to 400 ms with a maximum amplitude of approximately 3 μV. However, both groups showed a decreased amplitude and a weakening of the strength of the P200 as trials containing the standard stimulus progressed. By the end of the trial, the P200 appeared at only approximately 200 to 300 ms. At this time, the MCI group exhibited a more blurred and scattered P200, which reflects a greater degree of weakening than the HC group. In the target TT plots of both groups, the N100 occurred at approximately 150 ms with a maximum amplitude of approximately −5 μV, and the P300 component, including the P200, occurred at approximately 200 to 450 ms with a maximum amplitude of approximately 5 μV. In contrast to the standard TT plot, both groups showed little decrease in ERP amplitude or strength with increases in the number of target stimuli trials.

## 4 Discussion

In this study, we analyzed ERP signals obtained from thousands of elderly participants in clinical study who were subdivided into the CN, SCD, aMCI, and naMCI subgroups. Specifically, we measured two-channel prefrontal ERP signals using a portable, convenient and fast method, which is well suited for use with older adults experiencing cognitive decline. Pre-signal processing and ERP component detection algorithms specifically adapted for two-channel prefrontal ERP signals were performed. Subsequently, waveform, connectivity, TF and TT analyses of the ERP signals obtained in the standard and target epochs were conducted. We focused on the differences between groups, with a particular emphasis on signals in the beta band.

The 2 main groups significantly differed in age and sex but not education level. On cognitive function scales across various categories of neuropsychological measures, the MCI group scored significantly lower than the HC group. This indicates a decline in overall cognitive function. However, no significant differences were found in the severity of depression symptoms or activities of daily living between the groups. The mean difference analyses between the 2 main groups revealed that the MCI group had a lower number of correct responses and a higher error rate than the HC group in both the standard and target conditions. These findings align with the expected differences in cognitive function between the two groups, and they are consistent with the results of a previous study (Papadaniil et al., 2016). In addition, despite the consistent and prompt responses required in the task, the MCI group exhibited significantly higher variability in response time to target stimuli than the HC group. Within the HC group, SCD exhibited grater variability than CN, highlighting the differences between these two subgroups. In contrast to previous studies, which have reported significant differences in response time between HCs and individuals with dementia (Patterson et al., 1988; Toda et al., 1993), we did not find significant differences in response time in this study. This suggests that there was no substantial difference in stimulus evaluation, response selection, or response performance between the HC and MCI groups (Toda et al., 1993). However, it is worth noting that despite instructing both groups to respond as quickly and consistently as possible, we found that the MCI group exhibited significantly higher variability in response times to target stimuli than the HC group. This increased variability in response time may provide a more meaningful reflection of the differences among the 4 subgroups than the mean response times.

Regarding the P300 latency in the target condition, the MCI group tended to exhibit longer latencies than the HC group, although this difference was not statistically significant. However, the SD of the P300 latency was significantly larger in the MCI group than in the HC group. These findings indicate that individuals with MCI exhibited greater variability in the latency of the P300 component elicited by the 64 target trials, resulting in less consistent responses compared to those of the HC group. Furthermore, the percentile of the SD of the COH value was significantly higher in the MCI group than in the HC group in both the standard and target epochs. This indicates that the variability in synchronization between the left and right prefrontal lobes was greater in the MCI group than in the HC group. This could be interpreted as a lower phase angle consistency in the MCI group, suggesting a less stable and less consistent phase relationship between the prefrontal lobes.

As described above, the differences between the HC and MCI groups primarily involved differences in variability. In contrast, we did not find evidence of longer P300 latencies in the MCI group, nor did we identify this variable as a suitable biomarker, as has been reported in previous studies (Gironell et al., 2005; Bennys et al., 2007; Morrison et al., 2018). The differences in P300 amplitude between MCI patients and HC are particularly intriguing and have been the subject of conflicting reports. These discrepancies are likely due to variations in study design and measurement regions, as the oddball task used may not have required full cognitive capacity from MCI patients, potentially affecting the observed P300 amplitude. In this study, we focused on individuals with MCI rather than dementia, as MCI patients exhibit less pronounced cognitive decline than those with dementia. Additionally, the lack of observed P300 differences may be partially attributed to the generally smaller ERP responses in the prefrontal cortex compared to the occipital lobe. Furthermore, the oddball task used in our study may have been relatively easy for the MCI patients and might not have required full cognitive load.

ANCOVA revealed that the mean PLV in the positive time window after stimulus onset of the beta band for the standard epoch was significantly lower in the MCI group than in the HC group. This indicates that the phase synchronization over time since stimulus presentation was weaker in the beta band in individuals with MCI. However, no phase synchronization differences were observed in the other frequency bands (alpha and theta). The ANCOVA and *post hoc* comparisons among the 4 subgroups revealed significant differences between the CN and naMCI subgroups in terms of behavioral measures, specifically the SD of response time. Additionally, significant differences were observed between the CN and aMCI subgroups in terms of the SD of COH values for both the standard and target epochs. naMCI is characterized by declines in executive function and cognitive control (Corbo and Casagrande, 2022). Therefore, it was expected that the naMCI subgroup in this study would exhibit impairments in overall behavioral performance, such as responding to auditory stimuli and pressing buttons in the oddball task. On the other hand, aMCI primarily manifests as cognitive decline related to memory and is often associated with hippocampal damage. It also confers a higher risk of progression to AD (Golob et al., 2007; Li et al., 2017). These characteristics could impact brain connectivity and result in reduced synchronization between brain regions involved in memory processes (Yener et al., 2019). This could potentially explain the greater variability in COH values observed in the aMCI subgroup compared to the naMCI subgroup within the MCI group. Similarly, within the HC group, the SCD subgroup appeared to exhibit impaired memory-related functions compared to the CN subgroup.

In the grand average standard ERP waveform analysis, the N100 component was most prominent, followed by the P200 component. These findings align with those of previous studies (Oray et al., 2002; Morrison et al., 2018), and the waveforms of the HC and MCI groups were nearly identical. On the other hand, in the grand average target ERP waveform analysis, the P200 amplitude observed was slightly larger than the P300 amplitude. The P200 component naturally evokes during early sensory processing, resulting in a relatively consistent pattern across participants. However, the P300 component exhibits greater variability among participants due to differences in their cognitive functions. This could be considered one of the distinctive characteristics of ERP data obtained from the prefrontal lobe of older adults. The observed P300 tendency in the prefrontal lobe may slightly differ from many previous studies (Golob et al., 2002; Gironell et al., 2005) that primarily focused on the parietal lobe due to its distinct physiology and anatomy. It has also been reported that younger individuals often exhibit a more prominent P300 in the parietal or occipital lobe, while older individuals tend to display a more prominent P200 in the frontal lobe (McEvoy et al., 2001). The grand average target ERP waveform across the 4 subgroups revealed that the P300 component of the aMCI subgroup exhibited a notably longer latency than that of the other three groups. The observation of a longer P300 latency in the MCI group compared to the HC group appeared to be driven more by the aMCI subgroup than by the naMCI subgroup.

In the grand average standard PLV waveform analysis, significant differences were observed across nearly all time ranges in the beta band. This finding suggests that the phase-amplitude coupling in the beta band between the left and right prefrontal lobes was impaired in the MCI group compared to that in the HC group. Additionally, no significant differences were found in the alpha and theta bands in any time range. This suggests that the MCI group experienced a loss of synchronization in the beta band, while other frequency bands remained unaffected (Handayani et al., 2018). In the grand average target PLV waveform analysis within the beta band, significant differences were found only within a narrow time range at the onset of the P200 and P300 components. This indicates that the inter-network connectivity of the MCI group was weaker than that of the HC group, specifically during the short time span corresponding to these two components, which were dominant in the target epochs. The analysis of the grand average standard and target PLV waveforms within the beta band across the 4 subgroups indicated that the CN subgroup exhibited the highest level of phase synchronization of neural activity in nearly all time windows, with stronger brain connectivity in the HC group compared to the MCI group. These findings suggest that the HC group, particularly CN more so than SCD, may process information faster and more efficiently between different brain regions than the MCI group. Conversely, the aMCI subgroup exhibited the lowest level of phase synchronization. Previous studies have applied quantitative EEG (QEEG) and reported a loss of beta band synchronization (Stam et al., 2003; Handayani et al., 2018), but none have specifically analyzed the PLV in the prefrontal lobe using ERP data. Additionally, none have conducted a detailed analysis of each response to standard and target stimuli in the oddball task.

In the grand average standard COH waveform analysis, significant differences were observed in the low and middle beta bands. This suggests that the phase relationship between the left and right prefrontal signals was weaker in the MCI group than in the HC group. Similar to the results of the PLV analyses, no significant differences were found in the alpha and theta bands. These findings indicate that the HC group exhibited a higher level of synchronization specifically in the beta band, suggesting that these individuals might exhibit more efficient information transfer than the MCI group (Gomez et al., 2009). Similarly, in the grand average target COH waveform analysis, higher COH values were observed in only the narrow middle beta band of the HC group compared to that of the MCI group. This finding indicates a stronger degree of synchronization in the beta band specifically in the HC group. The grand average standard and target COH waveform analysis across the 4 subgroups indicated that the CN subgroup exhibited the highest level of brain connectivity across nearly all frequency bands, while the aMCI subgroup demonstrated the lowest level of connectivity. The most consistent finding in prior studies on AD has been a reduction in COH values, specifically in the alpha band. However, these studies have primarily focused on QEEG data in brain regions other than the prefrontal lobe and have not reported any significant results in the beta band (Fonseca et al., 2011).

In this study, PLV analysis was conducted to explore ERP connectivity over time, and COH analysis was performed to investigate ERP connectivity over frequency. Interestingly, both analyses revealed similar trends. Brain connectivity plays a crucial role in cognition and information processing. Our findings indicate that brain connectivity was stronger in the HC group than in the MCI group. This suggests that the HC group may possess the ability to process information faster and more efficiently between different brain regions than the MCI group. Furthermore, since the oddball task used in this study is an experimental task designed to assess cognitive processing, the higher PLVs and COH values observed imply that the connectivity between brain regions involved in cognitive function might be enhanced. This is particularly relevant because neurophysiological diseases are commonly associated with disruptions in neural synchrony (Hogan et al., 2003). The observed lack of synchronization in the beta band between the two prefrontal ERP signals in individuals with MCI might contribute to the decline in cognitive function.

In particular, beta oscillations are frequently associated with sensorimotor processing and integration, as well as the regulation of cognitive states and sensorimotor control (Khanna and Carmena, 2017; Barone and Rossiter, 2021). Additionally, beta oscillations play a crucial role in maintaining stability in the motor system during periods of inactivity. Therefore, the observed significant differences in the beta band, coupled with the lack of differences in other frequency bands, imply that there might be marked distinctions in cognitive states and sensorimotor mechanisms between the HC and MCI groups. However, recent studies have indicated that the role of beta oscillations is unlikely to be limited to pure sensory or motor processes. Instead, beta oscillations have been implicated in a wide range of functions (Barone and Rossiter, 2021). Therefore, further research is needed to establish a causal role of beta oscillations in the sensorimotor system and to achieve a comprehensive understanding of their functional importance.

Additionally, the difference in brain connectivity or degree of synchronization between the 2 main groups was more pronounced during the standard epoch than during the target epoch. Specifically, significant differences were observed in brain connectivity or synchronization between the 2 main groups when responding to standard stimuli but not to target stimuli. Standard stimuli typically elicit a more predictable and consistent response, whereas target stimuli are designed to capture attention and evoke larger ERP responses (Barry et al., 2000). While ERP analysis can provide insights into basic responses to standard and target stimuli, connectivity analysis of these responses may offer greater insight into differences between the HC and MCI groups in specific aspects of brain function. In this context, the reduced ability of the MCI group to make predictions and maintain consistency with standard stimuli might have contributed to the observed lower connectivity characteristics. However, further research is needed to fully understand these dynamics and their implications. Furthermore, it is possible that the HC and MCI groups exhibited differences in the consistency of sensitivity to standard stimuli in the left and right prefrontal lobes (Aksu et al., 2022). The HC group may have exhibited more consistent sensitivity to standard stimuli while maintaining focus and anticipation of specific auditory cues. In contrast, the lower connectivity observed in the MCI group in response to standard stimuli could be attributed to inconsistent sensitivity in the two prefrontal lobes.

ERS is characterized by an increase in the intensity of brain waves within a specific frequency range that occurs for a limited duration, e.g., during an ERP. It signifies the synchronization of neural activity in response to a specific event (Pfurtscheller and Da Silva, 1999). Notably, ERS is closely linked to information processing and cognitive function (Krause et al., 2000), making it a valuable indicator of the brain’s response to specific events and useful for comprehending the underlying mechanisms of information processing. In the TF plots of ERSP values in this study, consistent trends of ERS were observed in the delta and theta bands from 150 ms onward, regardless of stimulus type. These findings imply that the HC and MCI groups have similar information processing mechanisms, indicating a comparable brain response to the oddball task.

On the other hand, ERD is observed in brain activity associated with cognitive or motor tasks and represents a form of brain preparedness (Vecchio et al., 2012). In simpler terms, ERD indicates that the brain is in a state of readiness to process and respond to a specific task. In the context of cognitive tasks, the presence of ERD suggests the suppression of brain activity related to the processing of cognitive stimuli (Dushanova et al., 2009). In this study, no ERD was observed in the middle beta band between 300 and 500 ms in the MCI group compared to the HC group. ERD refers to a decrease in the frequency and amplitude of brain activity during the time associated with a specific event or task. Our findings suggest that the brain’s information processing related to readiness and task performance in the beta band was not efficient in the MCI group as in the HC group. Specifically, among the subgroups, the aMCI subgroup exhibited minimal brain readiness in the beta band compared to the other groups.

ITC is a measure of the degree of synchronization of EEG signals across multiple trials and provides physiological insights into the level of signal transmission and synchronization between different brain regions (Basar et al., 2015). In the TF plots of ITC generated in this study, the HC and MCI groups exhibited nearly identical maps for the standard and target epochs, with a minor difference observed in only the alpha band. Consequently, from the perspective of information processing and cognitive function reflected by ITC, it is likely that the brain’s processing of auditory stimuli during the oddball task is similar in the HC and MCI groups.

In the TT plots of this study, a noticeable decrease in the amplitude and duration of the P200 component was observed with increases in the number of trials containing standard stimuli, while the N100 component remained relatively unchanged. Initially (during the first 30∼40 trials), the responses to the standard auditory stimuli were robust. However, as the trials progressed, there was a decline in ERP amplitudes within the P200 and early P300 time windows. This decline was more pronounced in the MCI group than in the HC group, as indicated by the paler colormap. Given that the P200 component is associated with early sensory processing in response to auditory stimuli, we could infer that early sensory and inhibitory processes are more impaired in the MCI group than in the HC group. On the other hand, the TT plot of the target stimuli exhibited a strong ERP response, while the N100 component appeared weaker than that in response to the standard stimuli. Additionally, the ERP amplitude within the P200 time window and the early part of the P300 time window demonstrated only a slight decrease as the trials containing the target stimuli progressed. This suggests that, unlike responses to standard stimuli, the effort and attentiveness of participants devoted to discriminating the target stimuli remained relatively stable throughout the trials. However, it should be noted that the MCI group displayed a slightly larger decrease in effort and attentiveness to the target stimuli than the HC group.

## 5 Limitations

Since this study focused on analyzing ERP data from only two channels of the prefrontal lobe, it was not possible to investigate the functions of other brain regions, such as the parietal, occipital, and temporal lobes. Furthermore, network analysis of brain regions using multiple channels was not feasible. Additionally, due to the limited availability of detailed previous studies analyzing the standard and target stimuli in the oddball task, it was challenging to make direct comparisons with and validate prior results. Nevertheless, the primary objective of this study, which was to analyze two-channel ERP data collected in the prefrontal lobe and compare the results of the HC and MCI groups, was adequately achieved. As we continue to collect follow-up data in our cohort, we anticipate being able to investigate disease progression in AD in the near future. Additionally, future research could benefit from a more comprehensive analysis that incorporates biological biomarkers, including APOE genotyping data.

## 6 Conclusion

The findings of the present study highlight the importance of considering variability and connectivity measures in cognitive decline research using two-channel prefrontal ERP signals evoked by standard and target stimuli. Differences between the two groups were not observed using conventional ERP component analysis; however, these differences were revealed through assessment of variability measures and connectivity analysis. The response of the MCI group to the target stimulus exhibited greater variability in response time and greater variability in the latency of the P300, leading to less consistent response performance compared to the HC group. In addition, the MCI group showed a loss of synchronization in the beta band (but not in other frequency bands) in response to standard stimuli, indicating a less efficient information transfer process. The observed differences in brain connectivity using the PLV and COH indices, particularly those in the beta band, may offer valuable insights into the mechanisms underlying cognitive impairment. Furthermore, the TF analysis revealed that information processing related to readiness and task performance (in the beta band) was less efficient in the MCI group, while the TT analysis indicated that early sensory and inhibitory processes were impaired to a greater extent in the MCI group than in the HC group. The aMCI subgroup exhibited high variability in COH values, leading to reduced synchronization between brain regions. In contrast, the naMCI subgroup showed impairments in their overall behavioral performance, characterized by declines in executive function and cognitive control.

This work contributes to the new perspectives of brain network of the early stages of cognitive impairment and the potential use of ERP connectivity measures in the beta band for assessing cognitive decline. We would like to emphasize that the results presented in this work are based on a large number of elderly participants that may be representative of Korean population and thus has high clinical power. Use of a simple and fast a few channel ERP measurement is increasingly promising as a screening method, either to replace or in combination with neuropsychological screening tests. This method offers the advantages of simplicity, portability, and identification of objective risk factors for cognitive decline and can be repeatedly used with minimal learning effects compared to questionnaire-based screening tools.

## Data Availability

The raw data supporting the conclusions of this article will be made available by the authors, without undue reservation.
