# Supplementary material for: Connectivity changes in two-channel prefrontal ERP associated with early cognitive decline in the elderly population: beta band responses to the auditory oddball stimuli
Source: Front Aging Neurosci. 2024 Oct 17;16:1456169. doi: 10.3389/fnagi.2024.1456169 (PMC11524914; doi:10.3389/fnagi.2024.1456169)
Supplement: Supplementary file 1 [file Table_1.docx]

Supplementary Material

# Connectivity changes in two-channel prefrontal ERP associated with early cognitive

**decline in the elderly population: beta band responses to the auditory oddball stimuli**

# Jang-Han Bae1,2, Minho Choi1, Jang Jae Lee3, Kun Ho Lee3,4,5, Jaeuk U. Kim1,6*

^1^Digital Health Research Division, Korea Institute of Oriental Medicine, Daejeon, South

Korea

^2^Aging Convergence Research Center, Korea Research Institute of Biotechnology (KRIBB),

Daejeon, South Korea

^3^Asian Dementia Research Initiative, Chosun University, Gwangju, South Korea

^4^Department of Biomedical Science, Chosun University, Gwangju, South Korea

^5^Korea Brain Research Institute, Daegu, South Korea

^6^KM Convergence Science, University of Science and Technology, Daejeon, South Korea

# * Correspondence:

Jaeuk U. Kim

[jaeukkim@kiom.re.kr](mailto:jaeukkim@kiom.re.kr)

# Keywords: Mild cognitive impairment, Event-related potential, Two-channel prefrontal

**EEG, Brain connectivity, Synchronization, EEG beta band, Auditory oddball paradigm**

A 0.1~30 Hz finite impulse response bandpass filter was applied to reduce noise

components in the high frequency and low frequency band (Widmann et al., 2015).

Considering the interstimulus interval of the task in this study, epoching was performed at -

200 to 700 ms from stimulus onset, and baseline correction was applied via constant trend

removal based on the average baseline voltage over a 200 ms window prior to stimulus onset.

Regarding behavioral measures, we judged each epoch as containing an error if the button

was not pressed at the appearance of the target stimulus, pressed prematurely (before target

stimulus onset), pressed at the appearance of the standard stimulus, or pressed multiple times

within one epoch. The following steps were performed in only the correct epochs (after

rejecting the epochs that were determined to contain response errors). Participants with an

error rate of 30% or higher across all epochs were considered to exhibit poor performance in

the oddball task, and their data were excluded from subsequent analysis.

In this study, we first set a threshold of 100 µV to remove noise and then deleted each

epoch that contained values above the threshold. Second, an algorithm for calculating

statistical outliers was applied using equations (1) and (2). We removed single epochs with

high variability relative to the variability of all epochs.

Outlier_low = Q1SD – 1.5 X IQRSD (1)

Outlier_high = Q3SD + 1.5 X IQRSD (2)

where Q1SD and Q3SD are the first and third quartiles of each epoch's SD values, and IQRSD is

the interquartile range of SD values. Epochs with SDs less than Outlier_low or greater than

Outlier_high were discarded. If at least 70% of the epochs were retained after these steps,

further analyses were performed; otherwise, the data from that participant were excluded. If

any of the standard or target ERPs were excluded from the analysis, the remaining ERP data

were also excluded from the analysis. Since each denoised ERP dataset may have a different

signal-to-noise ratio due to variation in the number of retained epochs, it was necessary to set

a uniform signal-to-noise ratio by equating the number of epochs. In this study, we randomly

selected 70% of the total epochs (180 standard epochs and 45 target epochs), as this number

followed the guidelines of a previous study (Boudewyn et al., 2018). The final randomly

selected epochs were averaged to obtain the average left and right ERP waveforms for each

participant, which were then merged to create a representative average ERP waveform.

**TABLE S1** Behavioral measures, ERP components and connectivity variables considered in

this study

| Variable | Unit | Description |
| --- | --- | --- |
| Behavioral measures |  |  |
| Cor_no | - | Number of correct responses |
| Err_rate | % | Error rate |
| RT_mean^a^ | ms | Mean response time |
| RT_std^a^ | ms | Standard deviation of response time |
| ERP components |  |  |
| N100_lat | ms | Latency of the N100 component |
| N100_amp | µV | Amplitude of the N100 component |
| P200_lat | ms | Latency of the P200 component |
| P200_amp | µV | Amplitude of the P200 component |
| P300_lat | ms | Latency of the P300 component |
| P300_lat_std | ms | Standard deviation of the P300 latency across trials |
| P300_amp | µV | Amplitude of the P300 component |
| P300_amp_std | µV | Standard deviation of the P300 amplitude across trials |
| P300_amp_mean | µV | Mean amplitude in the P300 time window |
| Connectivity |  |  |
| PLV_max_f | - | Maximum phase locking value in the f band (f = alpha, beta, or theta) |
| PLV_time_f | Ms | Corresponding time at which PLV_max_f occurred |
| PLV_P200_f | - | Mean phase locking value in the P200 time window in the f band |
| PLV_P300_f | - | Mean phase locking value in the P300 time window in the f band |
| PLV_P_f | - | Mean phase locking value in the positive time window in the f band |
| COH_max_f | - | Maximum coherence value in the f band (f = alpha, beta, or theta) |
| COH_freq_f | Hz | Corresponding frequency at which COH_max_f occurred |
| COH_mean_f | - | Mean coherence value in the f band |
| COH_std_f | % | Percentile of standard deviation of coherence values in the f band |
| COH_mean^b^ | - | Mean coherence value across all frequency bands |
| COH_std^b^ | % | Percentile of standard deviation of coherence values across all frequency bands |

^a^RT_mean and RT_std were computed in only the target epoch.

^b^Calculation of the COH_mean and COH_std was performed using the open source software

toolbox HERMES for MATLAB


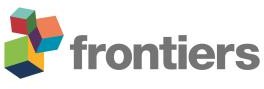


1. Widmann A, Schroger E, Maess B: **Digital filter design for electrophysiological data--a practical approach**. *J Neurosci Methods* 2015, **250**:34-46.
2. Boudewyn MA, Luck SJ, Farrens JL, Kappenman ES: **How many trials does it take to get a significant ERP effect? It depends**. *Psychophysiology* 2018, **55**(6):e13049.
